# Supplementary material for: Growth and Migration Blocking Effect of Nanaomycin K, a Compound Produced by Streptomyces sp., on Prostate Cancer Cell Lines In Vitro and In Vivo
Source: Cancers (Basel). 2023 May 10;15(10):2684. doi: 10.3390/cancers15102684 (PMC10216623; doi:10.3390/cancers15102684)
Supplement: Supplementary file 1 [file cancers-15-02684-s001.zip › cancers-2320230-supplementary/Supplementary Figure S1.pdf]

(A)

LNCaP

E-cadherin

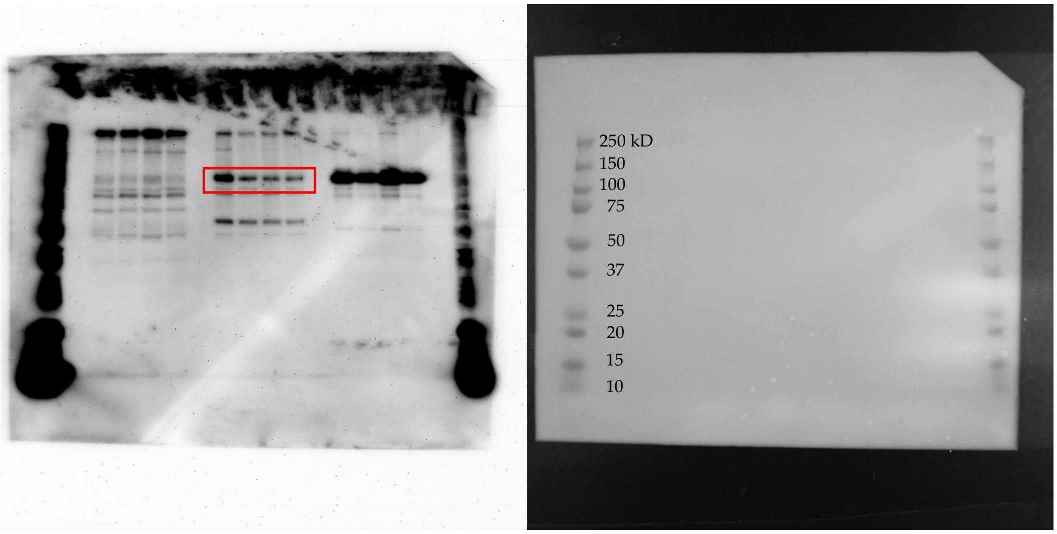

N-cadherin

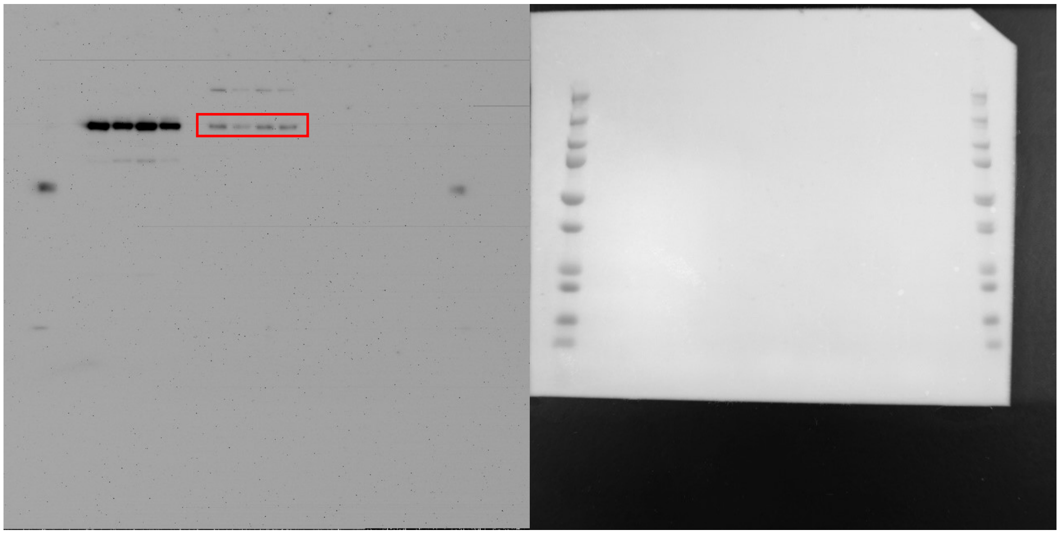

Vimentin

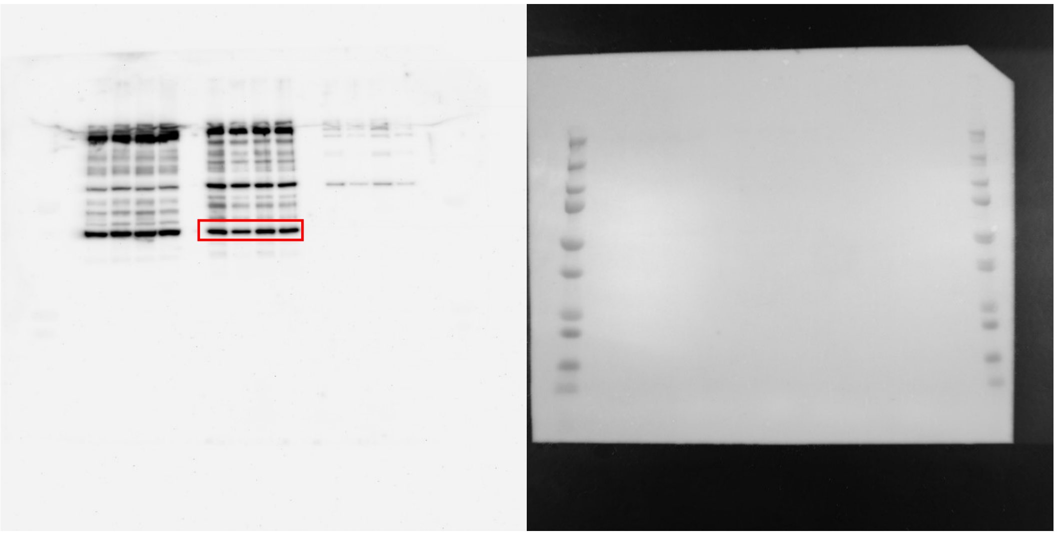

β-actin

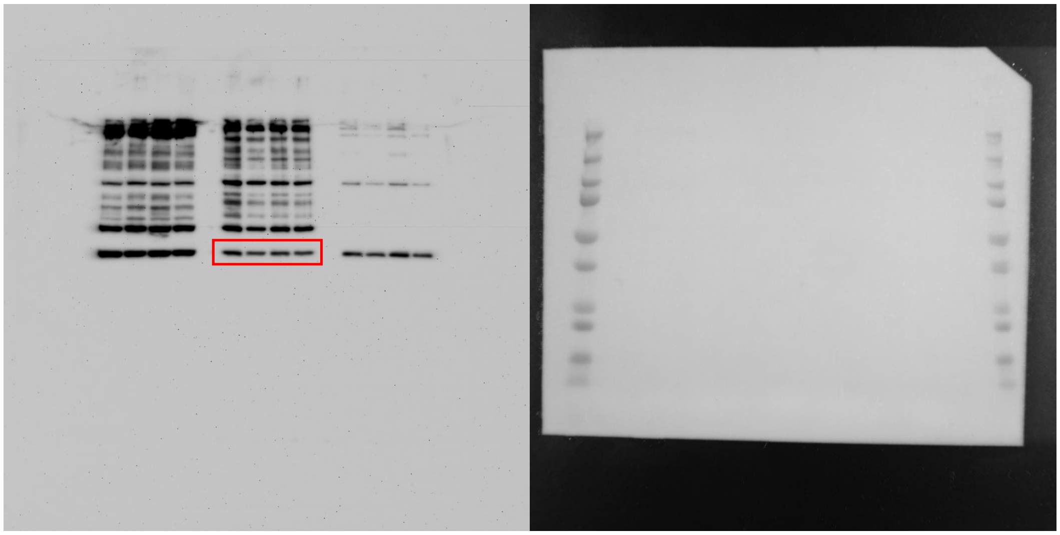

PC-3

E-cadherin

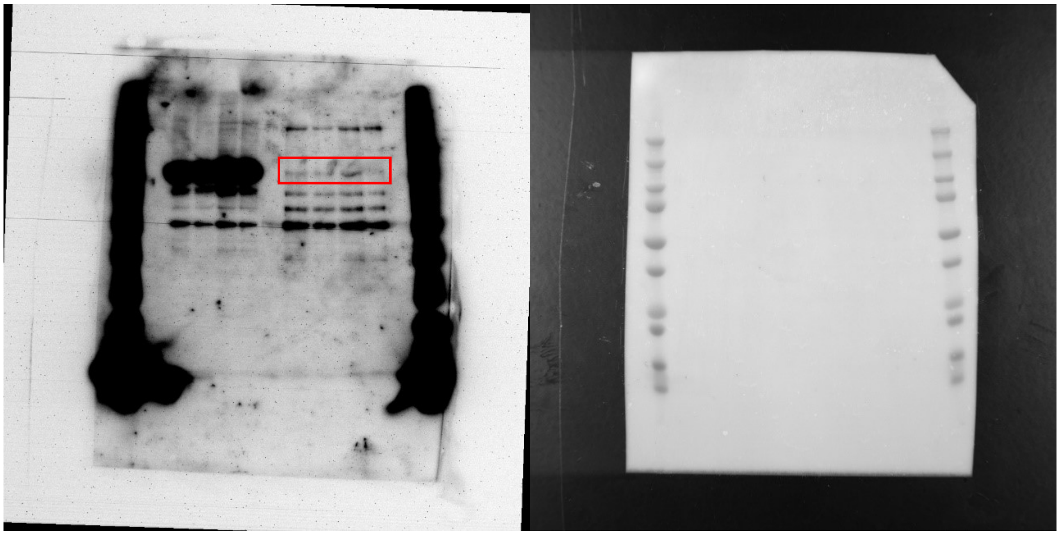

N-cadherin

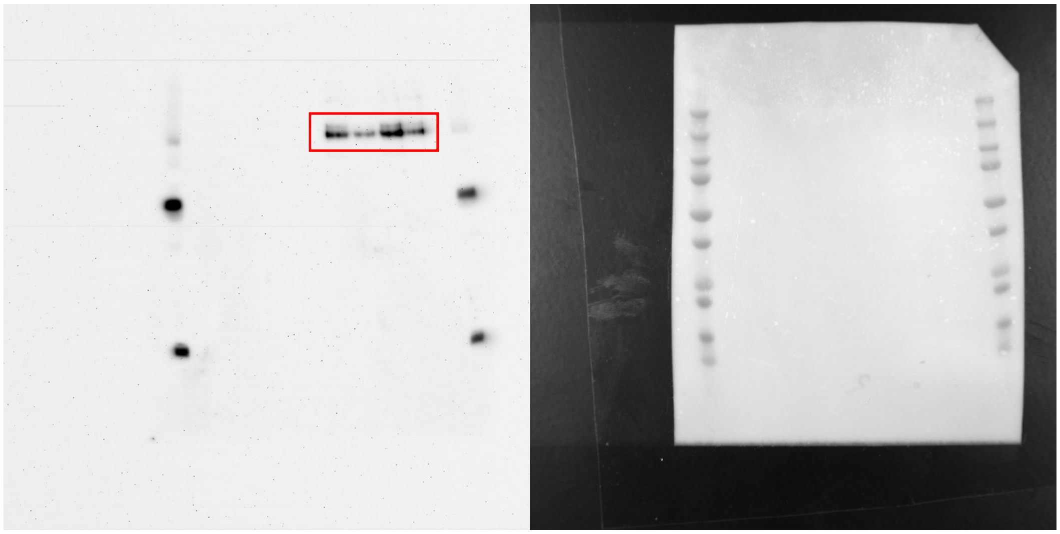

Vimentin

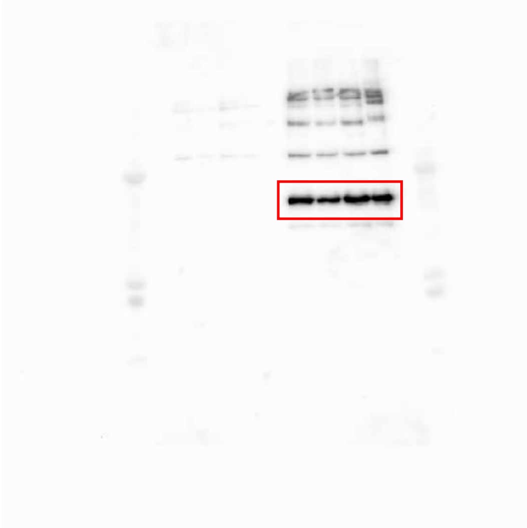

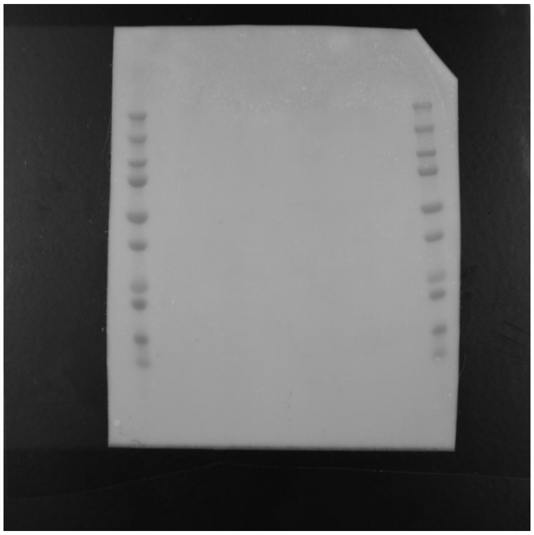

β-actin

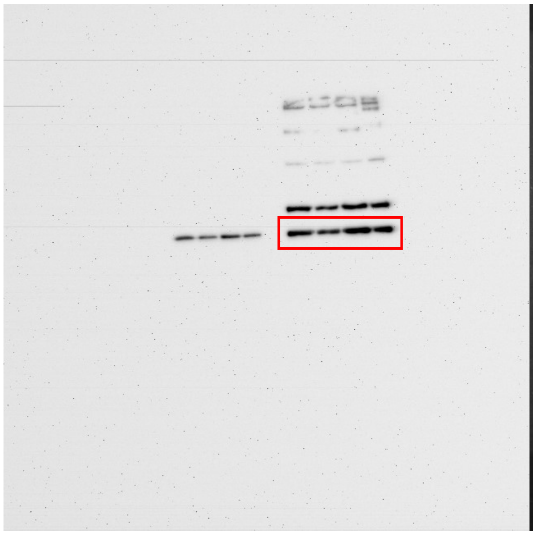

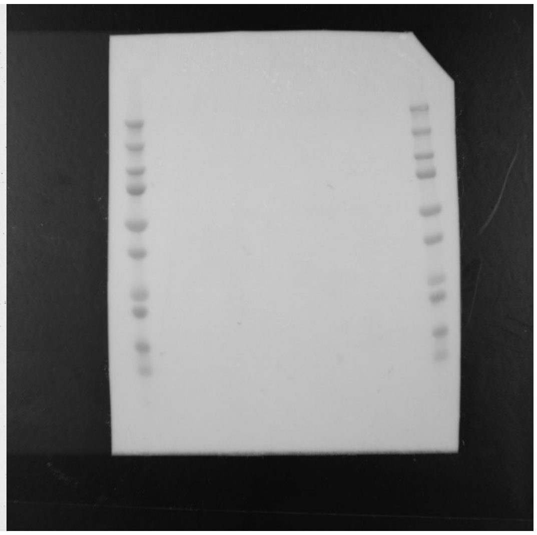

TRAMP-C2

E-cadherin

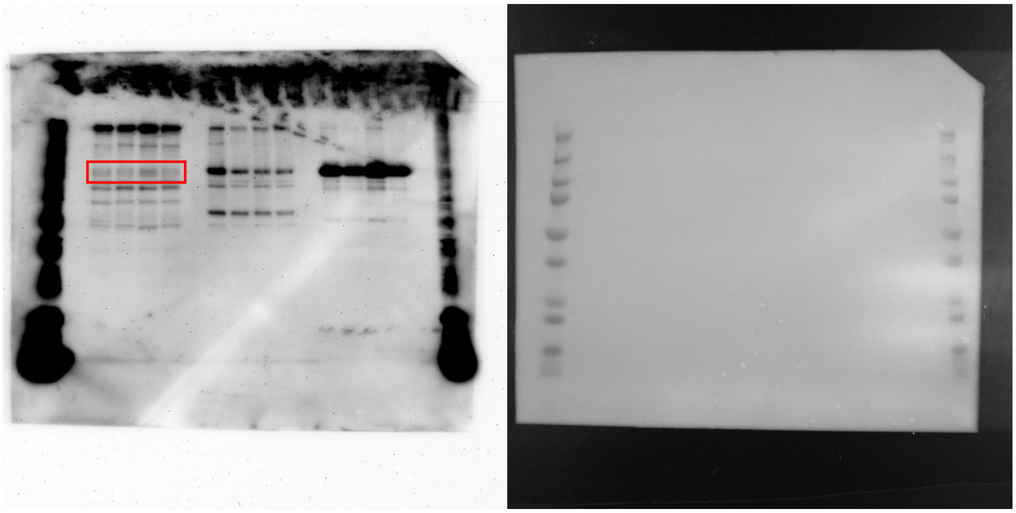

N-cadherin

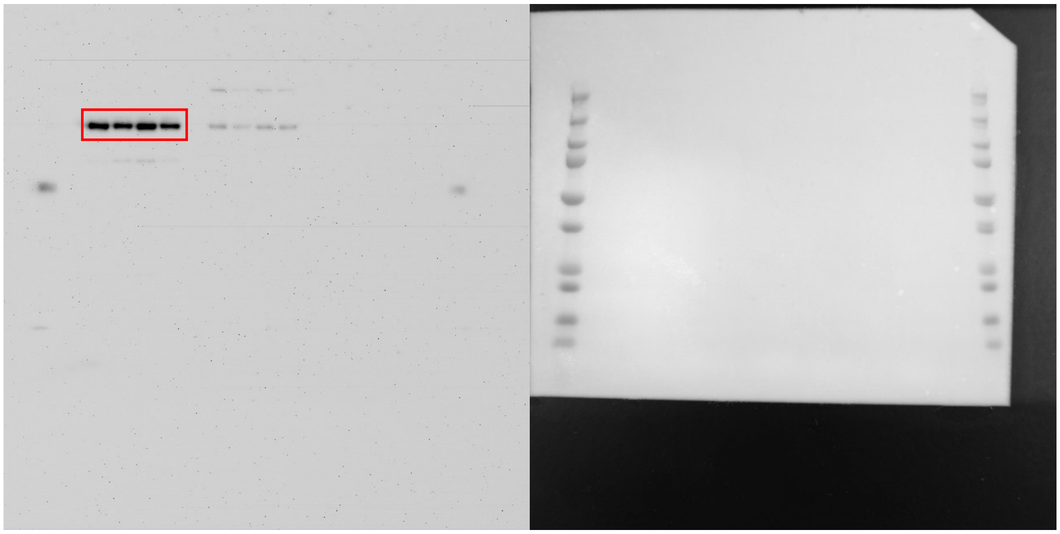

Vimentin

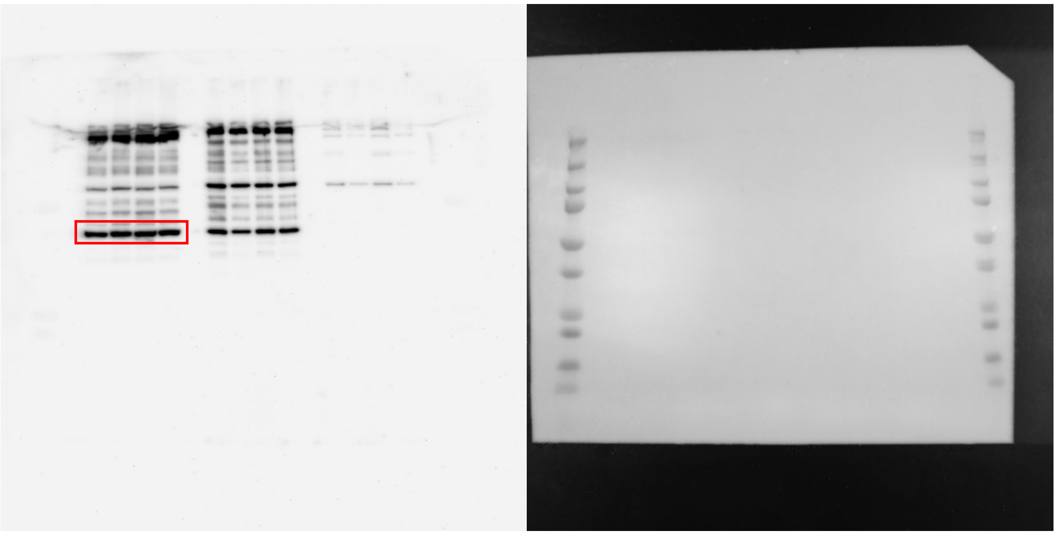

β-actin

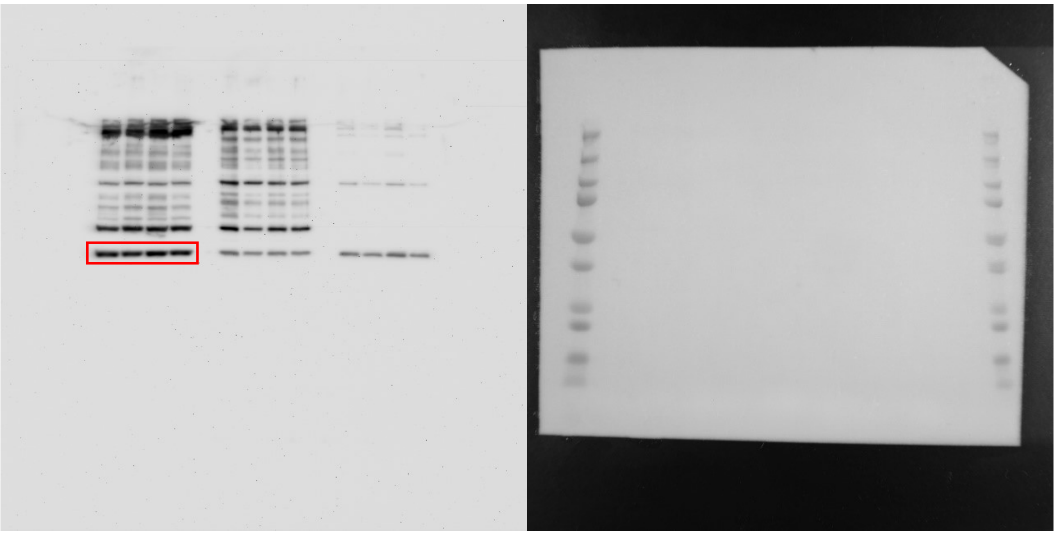

(B)

LNCaP

Slug

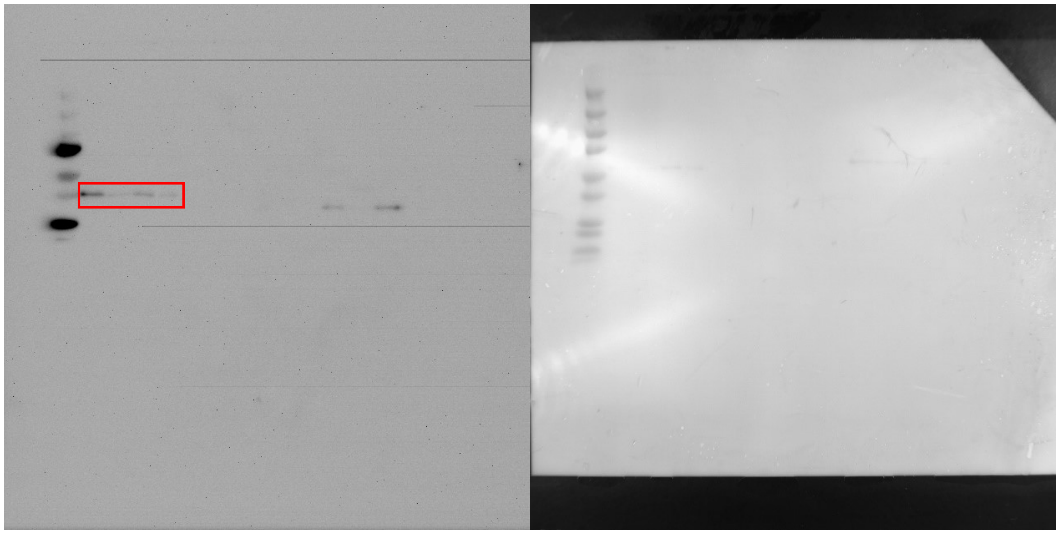

Snail

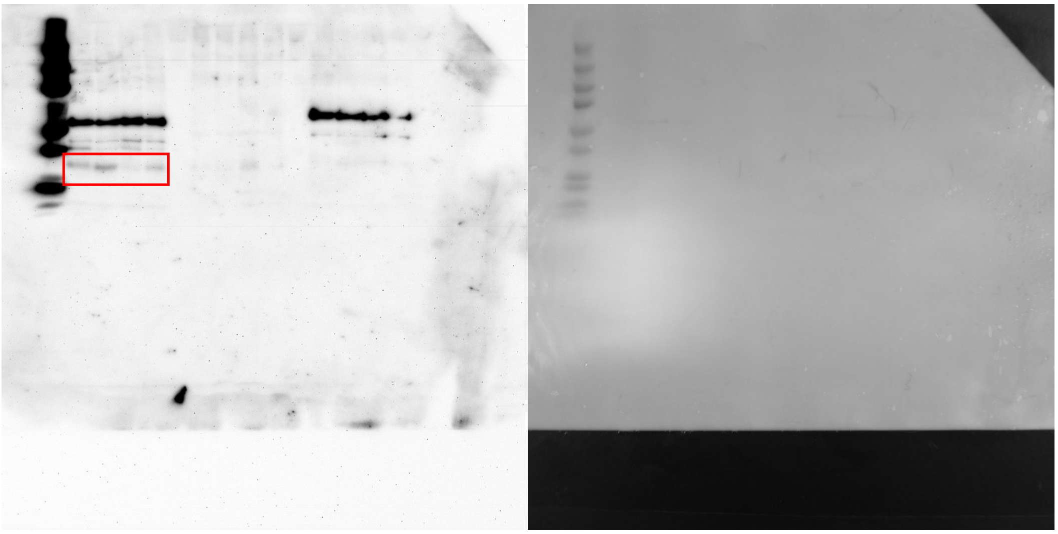

β-actin

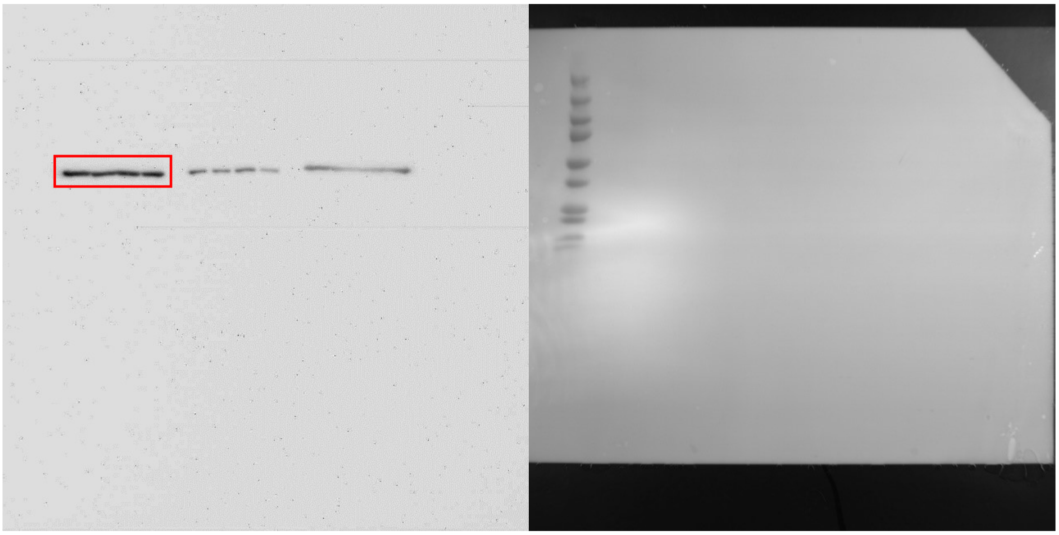

PC-3

Slug

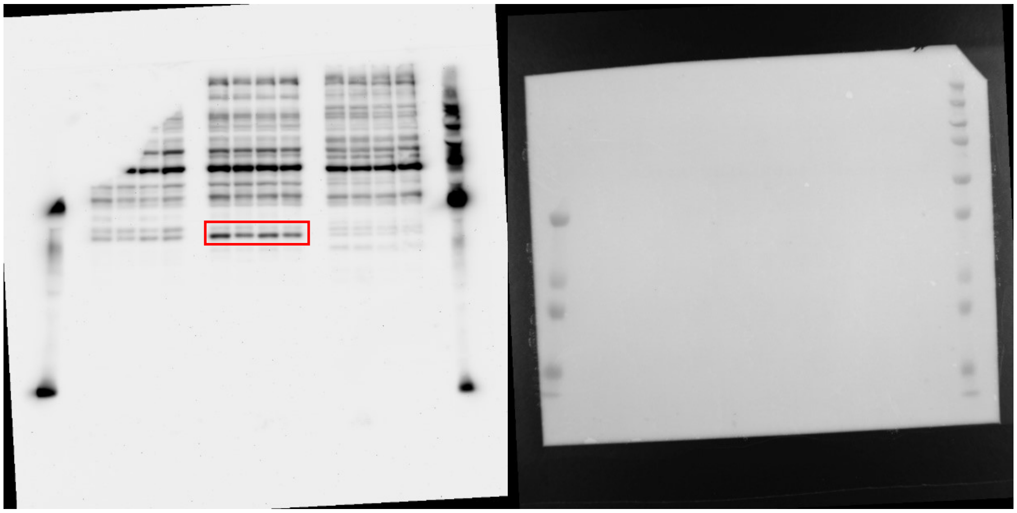

Snail

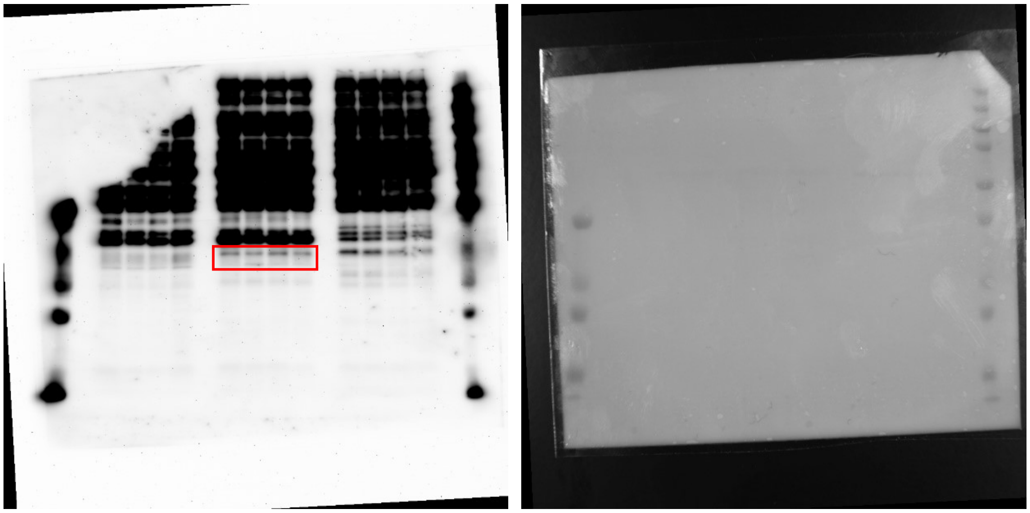

β-actin

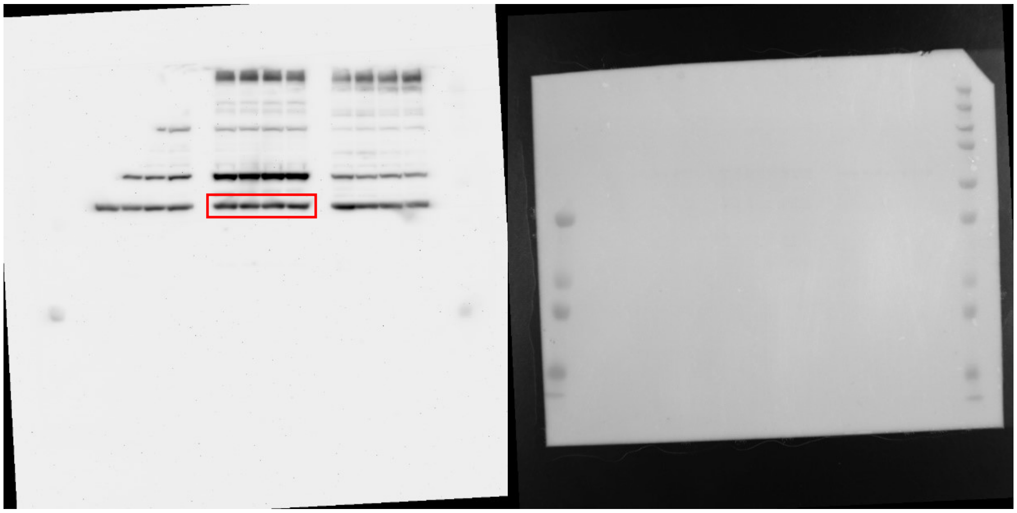

TRAMP-C2

Slug

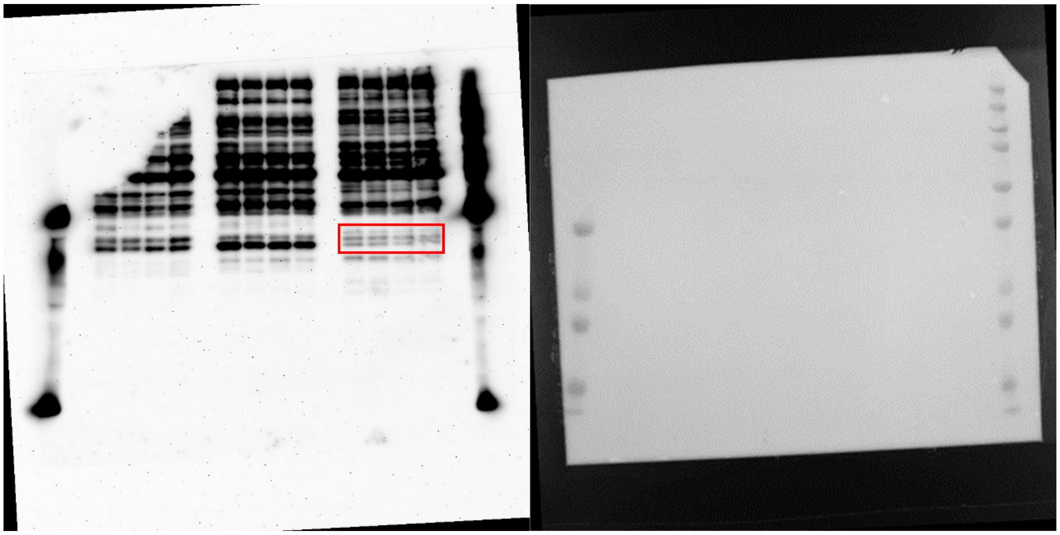

Snail

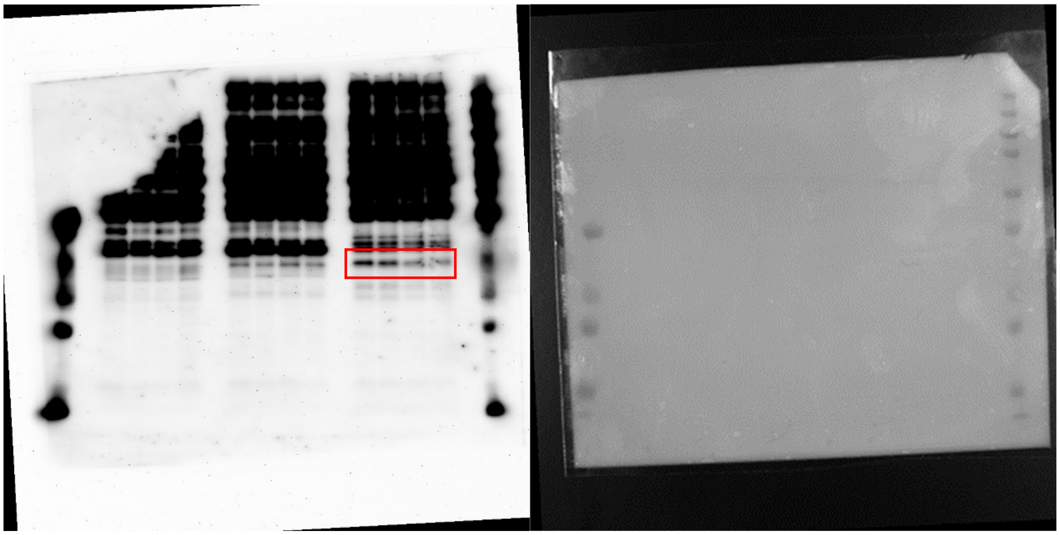

β-actin

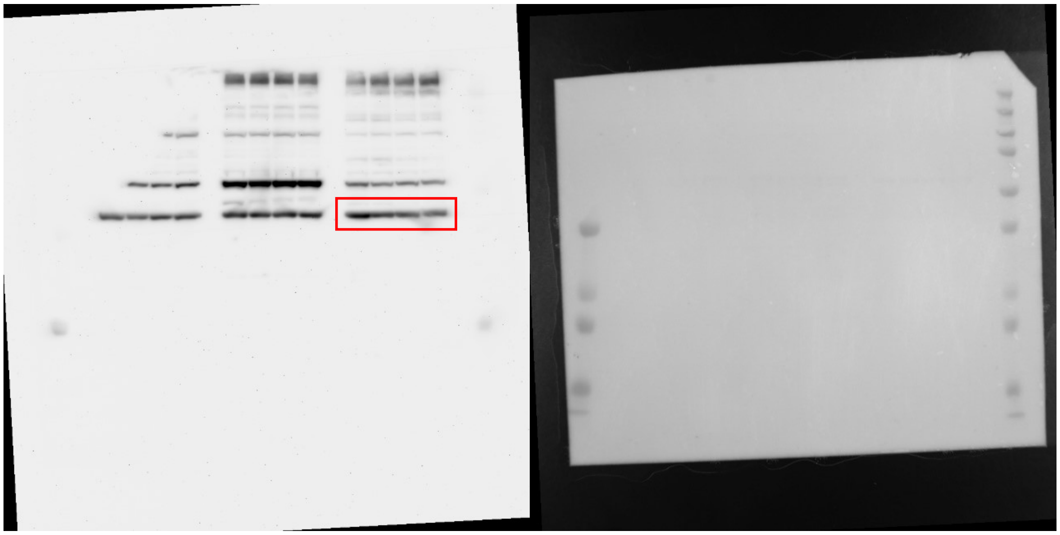

(C)

LNCaP

Phospho-p38

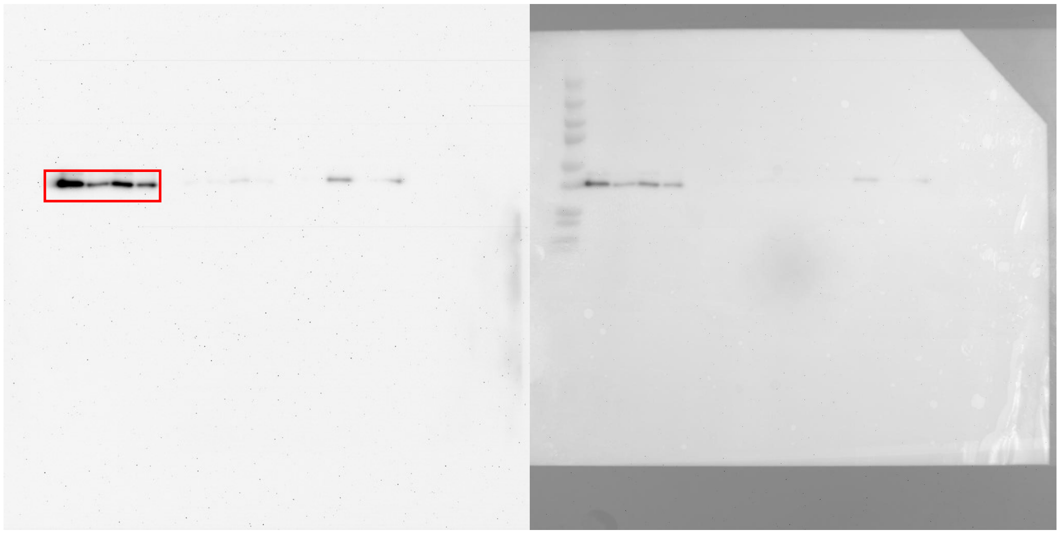

Phospho-SAPK/JNK

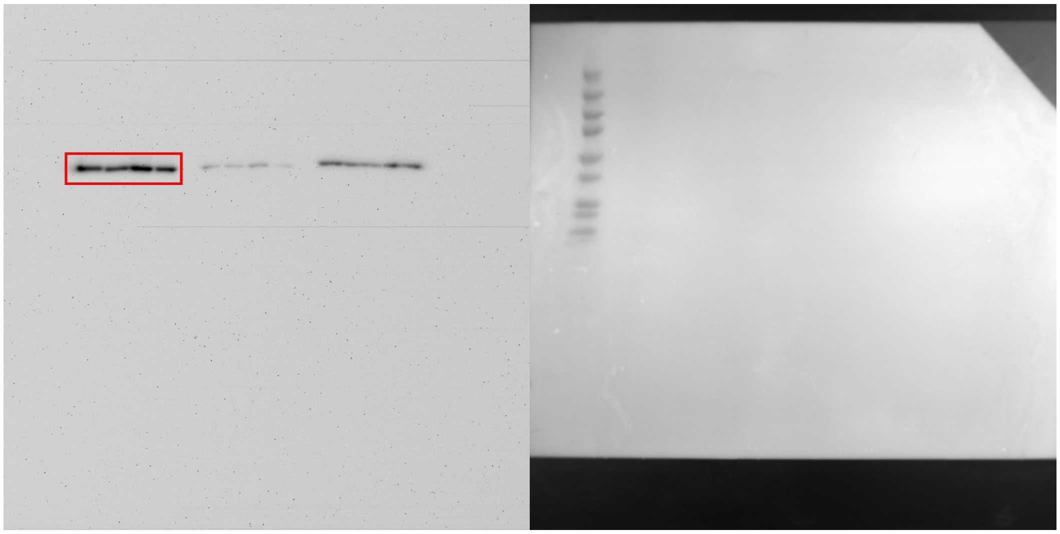

Phospho-Erk1/2

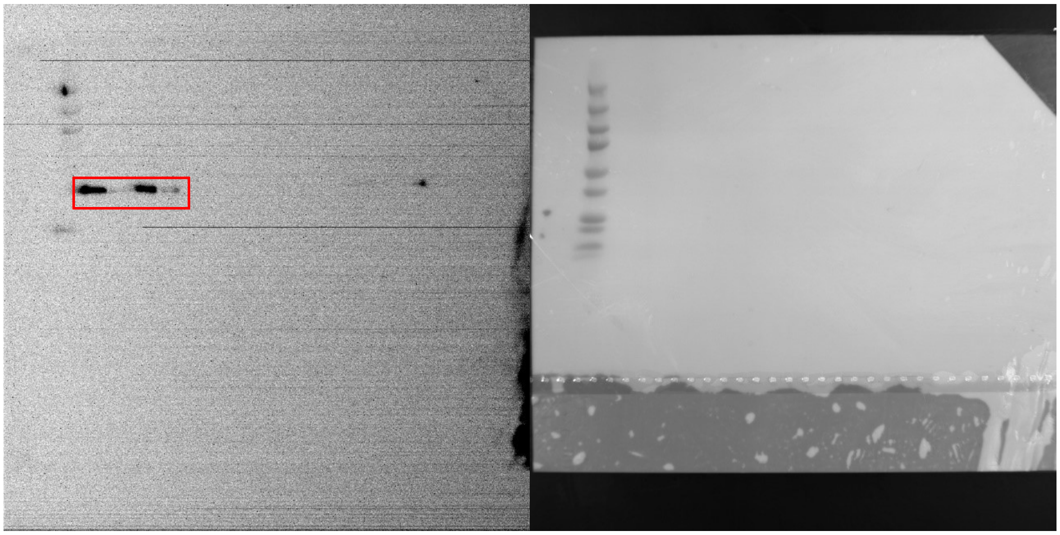

β-actin

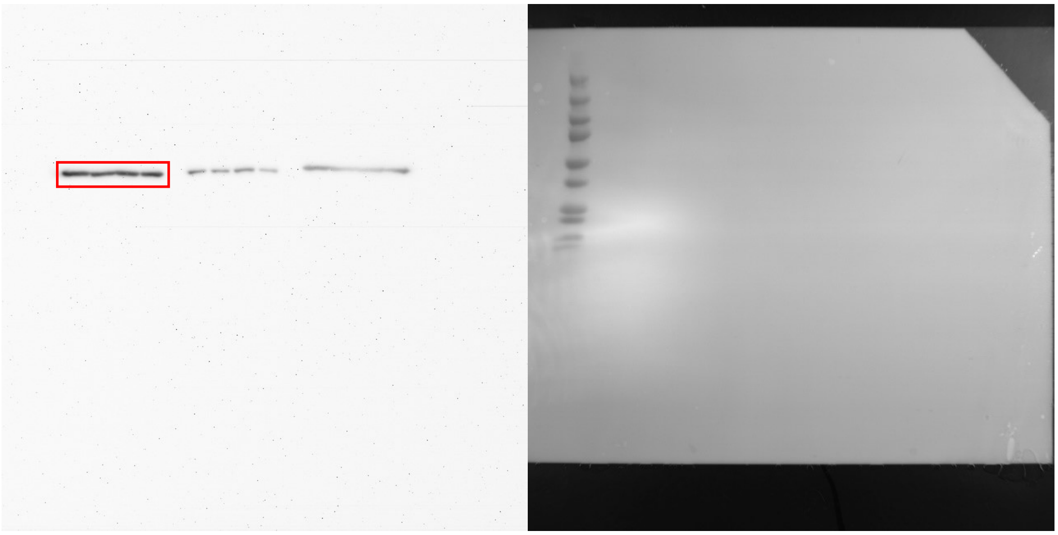

PC-3

Phospho-p38

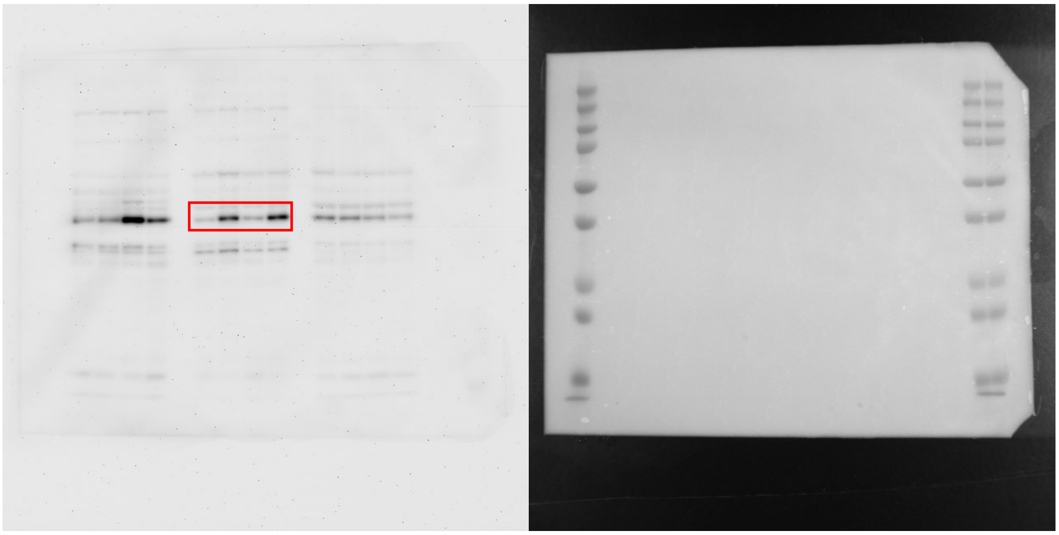

Phospho-SAPK/JNK

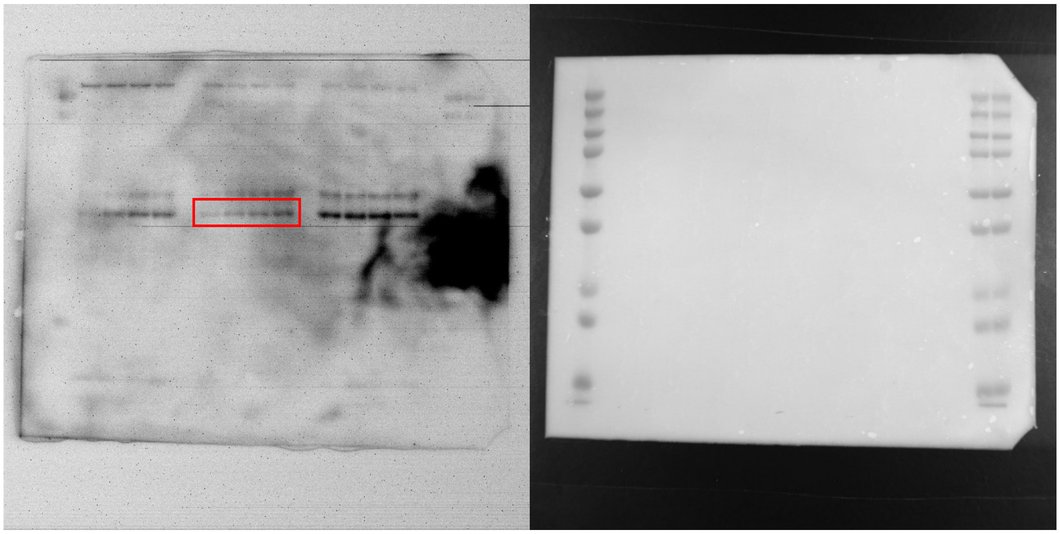

Phospho-Erk1/2

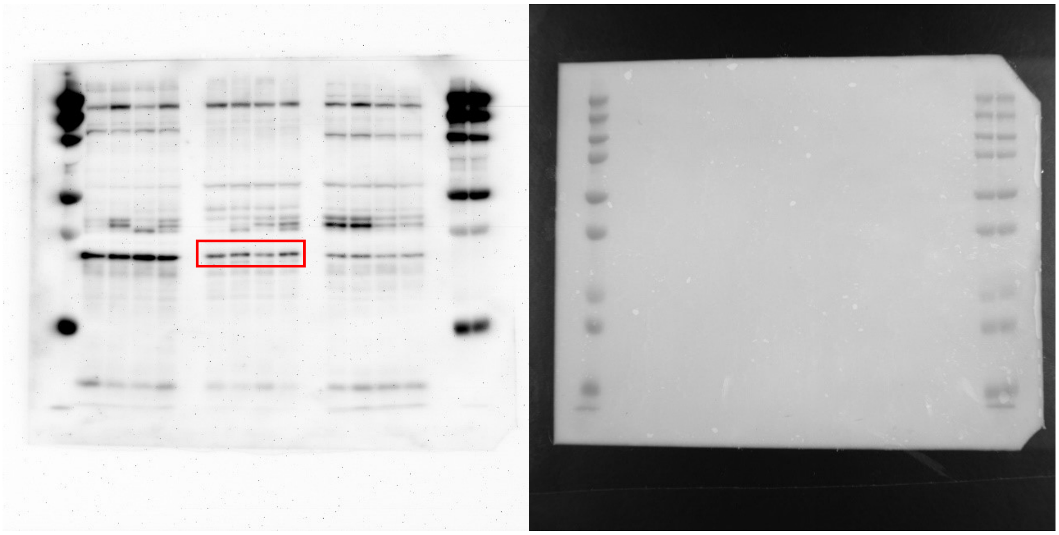

β-actin

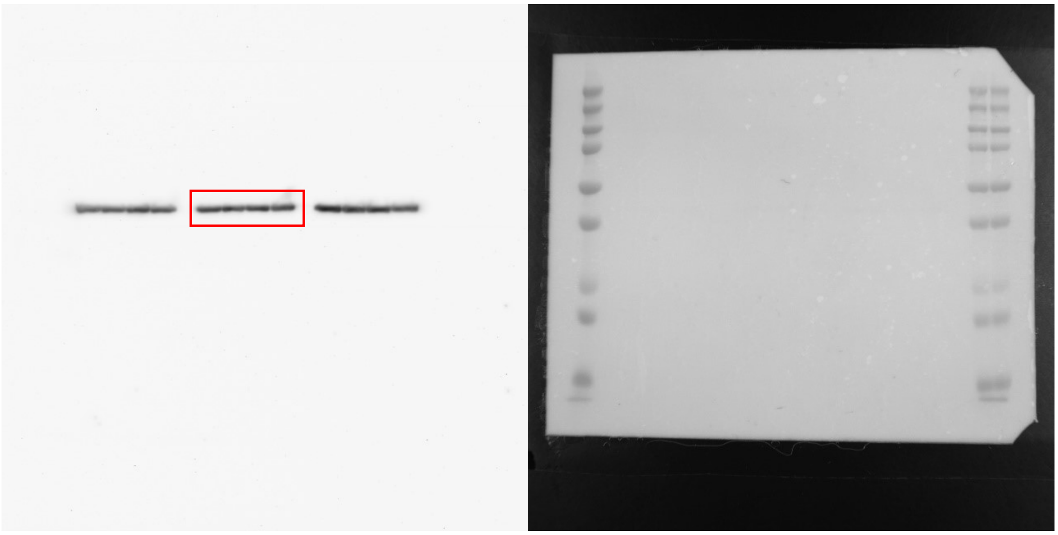

TRAMP-C2

Phospho-p38

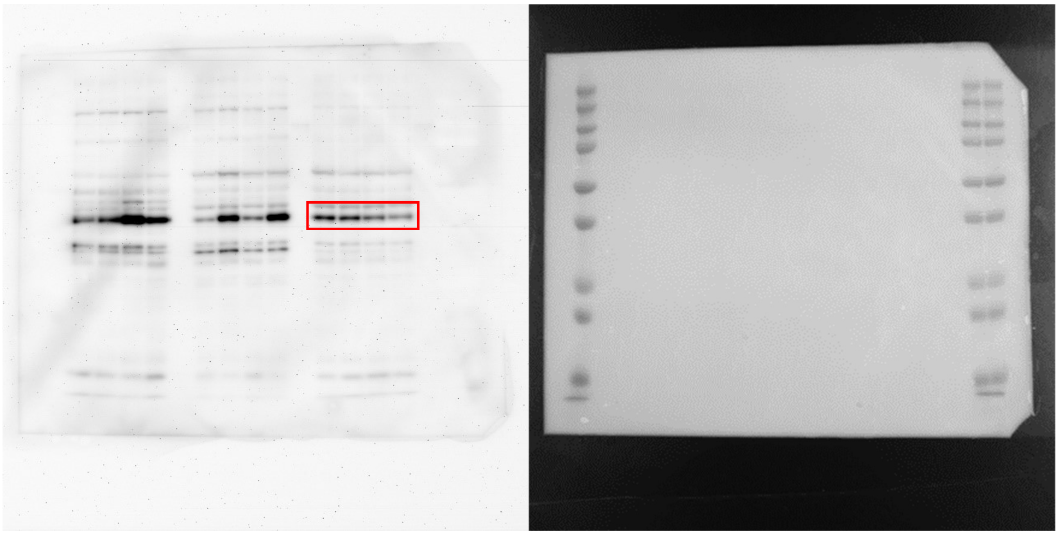

Phospho-SAPK/JNK

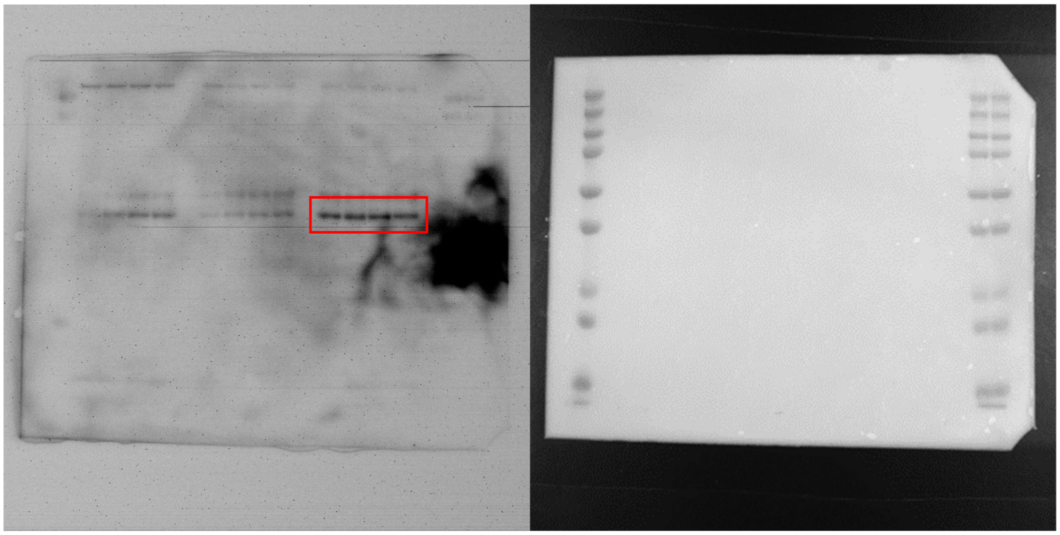

Phospho-Erk1/2

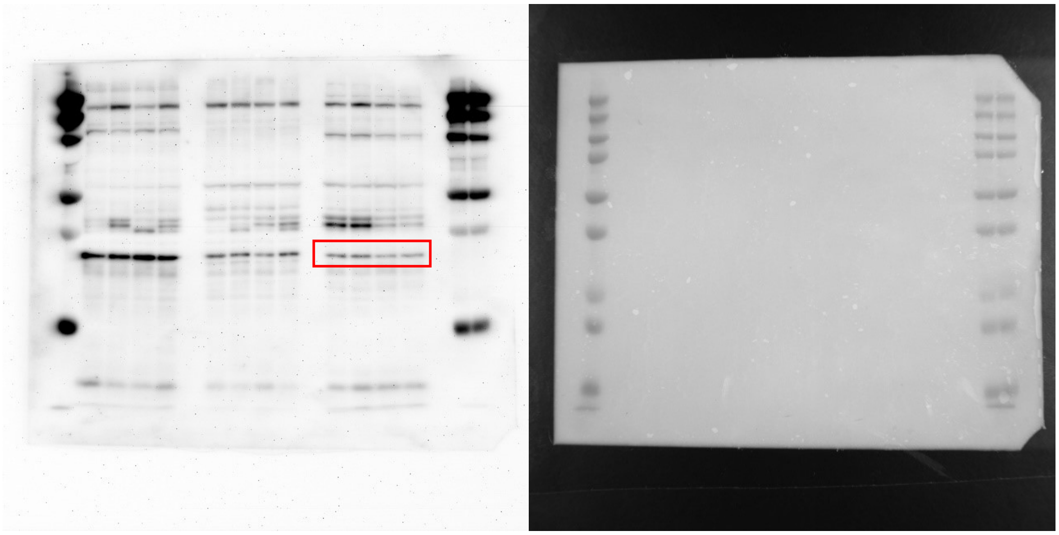

β-actin

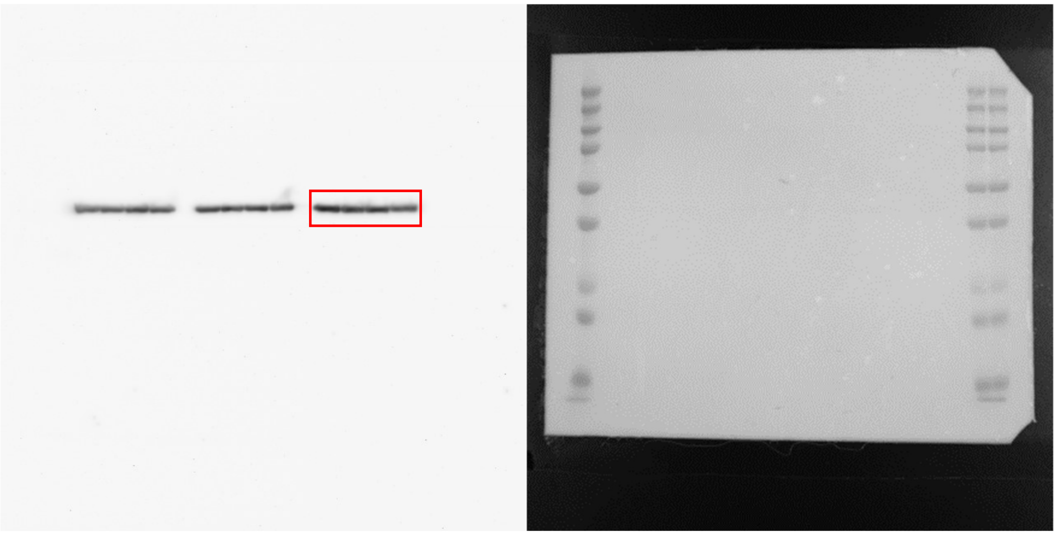

(D)

LNCaP

Ras

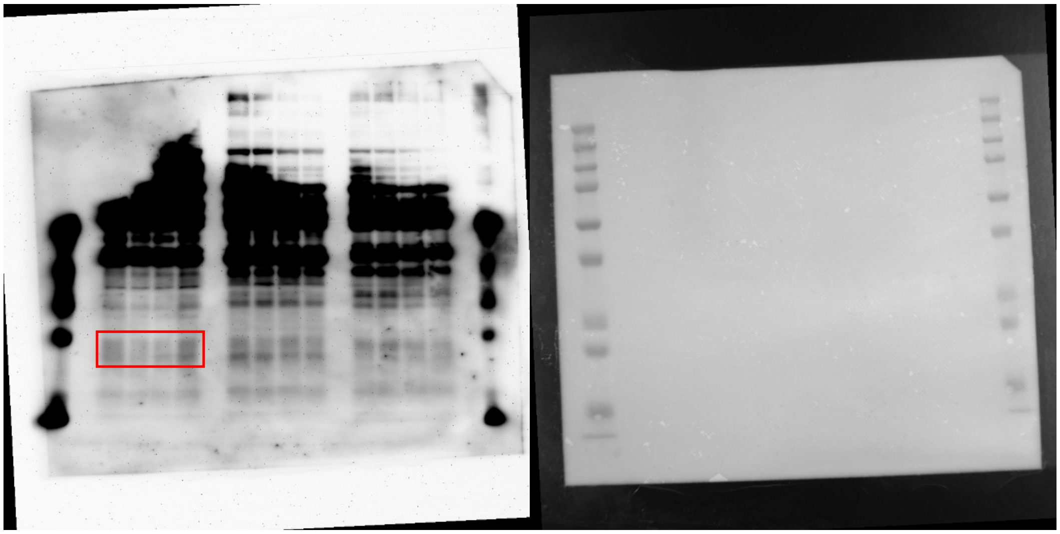

β-actin

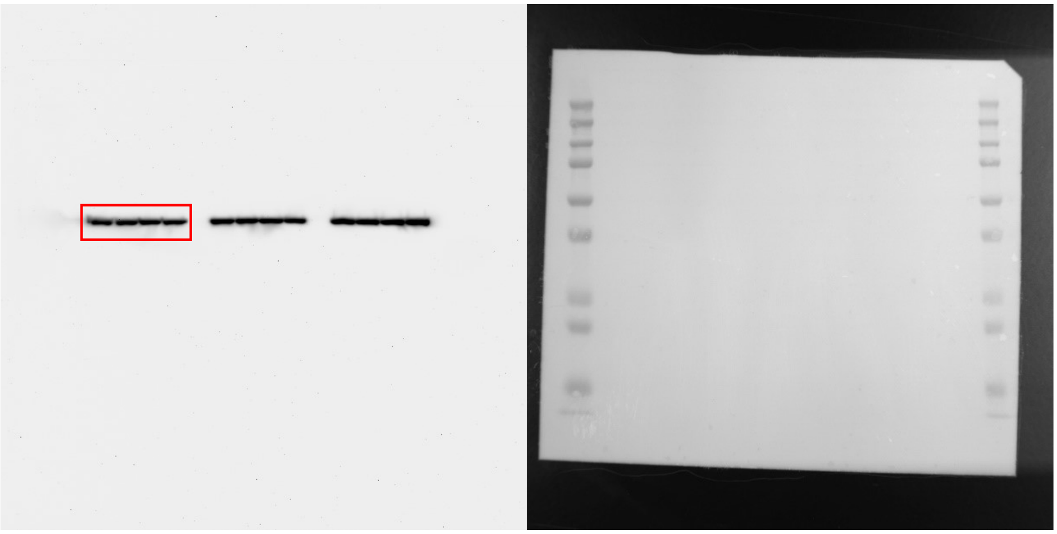

PC-3

Ras

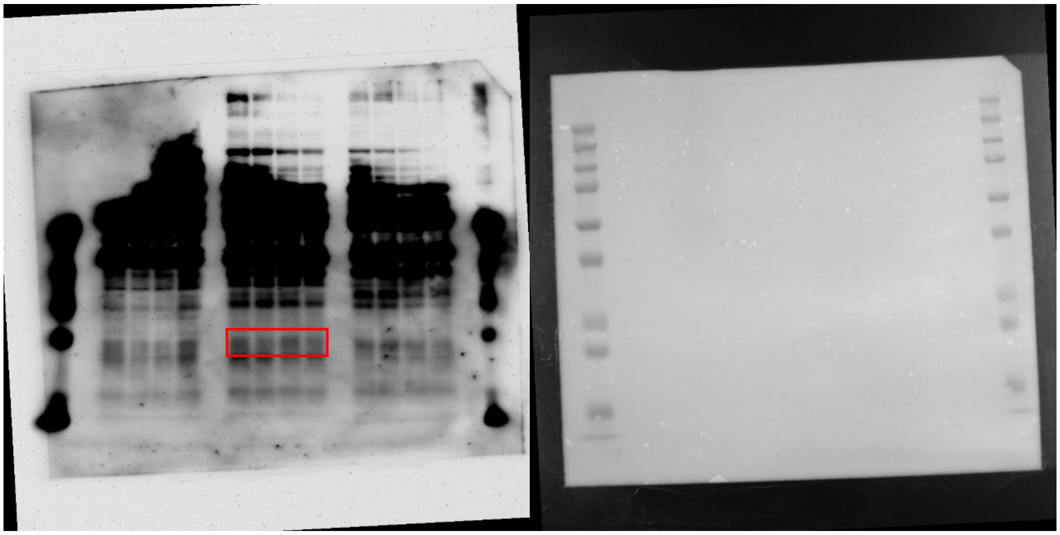

β-actin

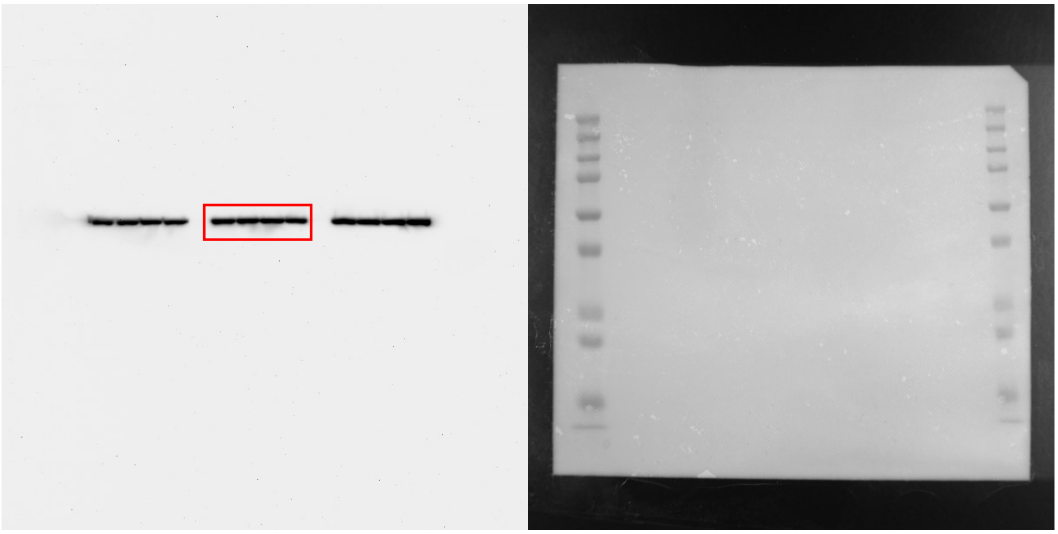

TRAMP-C2

Ras

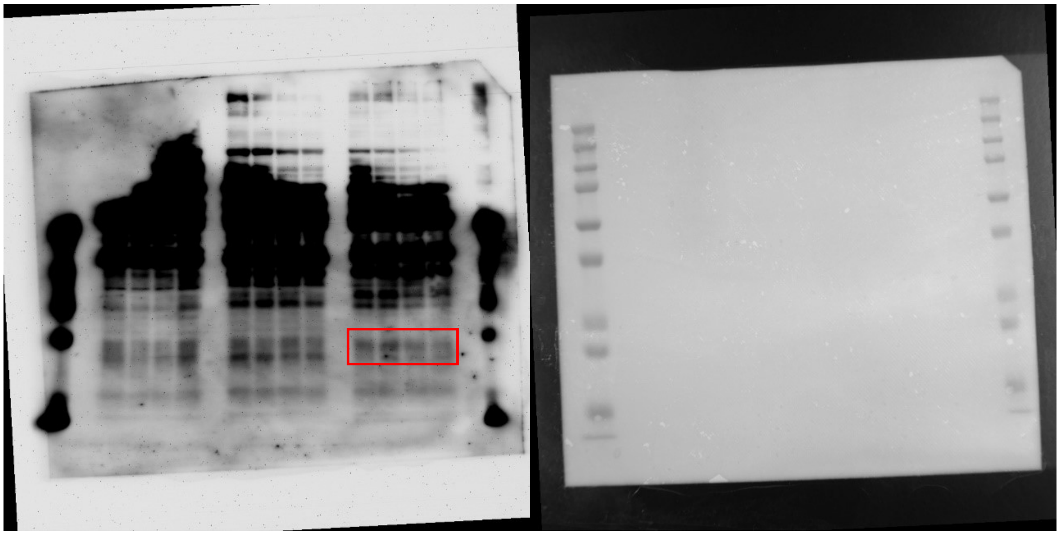

β-actin

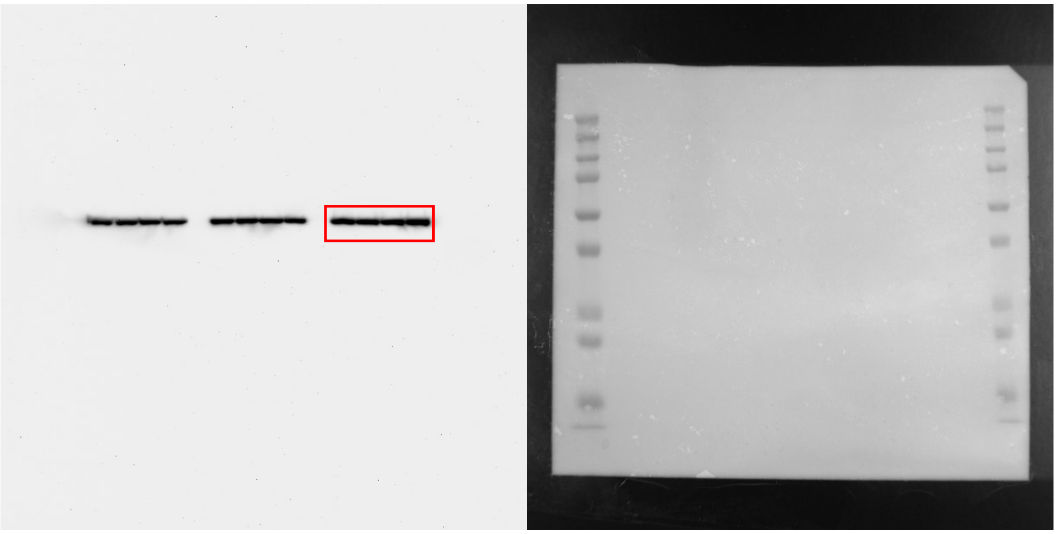

Supplementary Figure S1. Expression of EMT Related Protein and MAPK Signaling after Culture with Nanaomycin K. Protein expression of EMT-related markers and MAPK signaling. The expressions of (A)EMT markers (E-cadherin, N-cadherin, and Vimentin), (B)E-cadherin repressors (Slug, Snail), (C)MAPK signaling (phospho-p38, phospho-SAPK/JNK, phospho-ERK1/2), and (D)Ras were determined in the presence of 25 μg/mL Nanaomycin K and in the presence or absence of TGF-β in vitro for 48 h in LNCaP, PC-3, and TRAMP-C2 cells. β-actin was used as a housekeeping protein. In each protein, the left is a whole blot and

the right is a molecular weight marker taken at the same time. The molecular weight markers are all the same, and are shown from top to bottom as 250, 150, 100, 50, 37, 25, 20, 15, and 10 kD.
